# The metacaspase Yca1 maintains proteostasis through multiple interactions with the ubiquitin system

**DOI:** 10.1038/s41421-018-0071-9

**Published:** 2019-01-22

**Authors:** Amit Shrestha, Steve Brunette, William Lloyd Stanford, Lynn Arthur Megeney

**Affiliations:** 1 Ottawa Hospital Research Institute, Regenerative Medicine Program, Sprott Centre for Stem Cell Research, The Ottawa Hospital, Ottawa, ON K1H 8L6 Canada; 2 Department of Cellular and Molecular Medicine, University of Ottawa, Ottawa, ON K1H 8M5 Canada; 3 Department of Biochemistry, Microbiology and Immunology, University of Ottawa, Ottawa, ON K1H 8L6 Canada; 4 Ottawa Institute of Systems Biology, University of Ottawa, Ottawa, ON K1H 8M5 Canada; 5 Department of Medicine, University of Ottawa, Ottawa, ON K1H 8L6 Canada

**Keywords:** Protein aggregation, Ubiquitylation, Phosphorylation

## Abstract

Metacaspase enzymes are critical regulatory factors that paradoxically engage apoptosis and also maintain cell viability. For example, the *Saccharomyces cerevisiae* metacaspase Yca1 has been shown to be important for maintaining cellular proteostasis during stress, and the loss of this enzyme results in increased retention of aggregated material within the insoluble proteome. However, the molecular mechanism(s) by which Yca1 maintains cellular proteostasis remains unknown. Here, using proteomic analysis coupled with protein interaction studies we identified a direct interplay between Yca1 and the ubiquitin-proteasome system. We noted multiple ubiquitination sites on Yca1 and established Rsp5 as the candidate E3 ligase involved in this process. Further characterization of the ubiquitination sites identified the K355 residue on Yca1 as a critical modification for proteostasis function, managing both insoluble protein content and vacuolar response. We also identified a Yca1 phosphorylation site at S346, which promoted interaction with Rsp5 and the aggregate dispersal function of the metacaspase. Interestingly, proteomic analysis also revealed that Yca1 interacts with the ubiquitin precursor protein Rps31, cleaving the protein to release free ubiquitin. In turn, loss of Yca1 or its catalytic activity reduced the levels of monomeric ubiquitin in vivo, concurrent to increased protein aggregation. The K355 and S346 residues were also observed to influence the abundance of low-molecular weight ubiquitin. Together, these observations suggest that Yca1 maintains proteostasis and limits protein aggregation by ensuring a free flow of monoubiquitin, an essential precursor for ligase-enhanced Yca1 enzymatic activity and general proteasome-mediated protein degradation.

## Introduction

Metacaspases are functional orthologs of caspase enzymes and are grouped together into the clan cysteine dependent aspartic specific (C/D) family of proteases^1,2^. The yeast *Saccharomyces cerevisiae* possesses a single type I metacaspase known as Yca1 which has been shown to mediate apoptosis in yeast under various stresses^3^. However, like caspases, metacaspases also function in non-death scenarios, particularly to promote cell viability^4^. Yca1 was observed to be a regulator of cell cycle progression; the loss of which reduced cellular fitness and abolished the G2/M cell cycle checkpoint upon microtubule disruption^5^. Similarly, the *Leishmania* metacaspase, LmjMCA, has been shown to promote transit through the cell cycle, suggesting a conserved non-death activity^6^. Further investigation established that Yca1 exerted a pro-life effect by contributing to proteostasis maintenance. Specifically, Yca1 was shown to mediate key interactions with known members of the proteostasis network such as Cdc48, Hsp104, and the Hsp70/40 chaperone systems. Indeed, the loss of Yca1 led to increased retention of insoluble protein material within the cell, coupled with an engagement of the compensatory autophagic response^7^. Finally, Yca1 has been shown to limit/prevent protein aggregation in daughter cells during yeast aging studies^8^. A similar role has been described for the plant metacaspase AtMC1 suggesting that proteome regulation is a conserved metacaspase function^9^. Despite compelling observations that link Yca1 to protein aggregate management, the essential molecular controls that guide this physiologic function remain unknown.

Given the central role of Yca1 in managing protein aggregation and dissolution it is reasonable to assume that this metacaspase may collaborate with or integrate other proteostasis regulatory elements. For example, the ubiquitin proteasome system (UPS) is a well characterized cellular machinery that determines protein fate and function within the cell. Ubiquitin is a small highly conserved protein that is conjugated onto lysine (K) residues on substrates via a series of enzymatic steps. First, ubiquitin is activated by E1 ubiquitin-activating enzymes, which leads to its conjugation to E3 ligases via E2 ubiquitin-conjugation enzymes. Then, ubiquitin-conjugated E3 ligases interact with the substrate and facilitate the transfer of the ubiquitin moiety^10,11^. Within the cell, ubiquitin is expressed as a ribosomal fusion protein which is required to undergo cleavage for the production of mature ubiquitin^12^. Rps31 is one such precursor which has been shown to be critical for ribosome biogenesis and cell survival^13^. Additionally, Rsp5 belongs to the homologous to E6-AP carboxy terminus (HECT) family of E3 ligases which directly catalyze the transfer of ubiquitin to the substrate^14^ compared to really interesting new gene (RING) E3 ligases which ubiquitinate substrates indirectly^15^.

The ubiquitination process is ATP dependent and can be repetitive, leading to the formation of ubiquitin chains anchored onto the N-terminal methionine or K residues, forming various types of chains with many linkages^16^. Polyubiquitin chains on substrates formed via K48 linkage are marked for proteasome degradation whereas, substrates with K11 and K29 chains are destined for degradation via the ERAD and the endosome respectively^17,18^. Tagging via K63 linked chain is non-degradative and associated with protein complex assembly in immune response signaling^19^. Alternatively, monoubiquitination induces conformational change to alter protein function^20^. Although mechanisms that regulate metacaspase function have not been discovered, it is reasonable to conjecture that these proteases may also be regulated via ubiquitin modification.

Here, using immunoprecipitation coupled with proteomics we identified an interplay between Yca1 and the UPS. We observed that Yca1 is ubiquitinated at multiple lysine residues, one of which (K355) oversees its protein regulatory role. Abrogation of this modification led to the altering of proteostasis safeguards within the cell. We further identified Rsp5 as the candidate E3 ligase which acts on Yca1 via an upstream phosphorylation site (S346). Notably, we identified Rps31 as a substrate for Yca1, suggesting that this metacaspase regulates proteostasis (in part) by stimulating ubiquitin biogenesis.

## Results

### Yca1 interacts with components of the ubiquitin protease system (UPS)

**Fig. 1:**
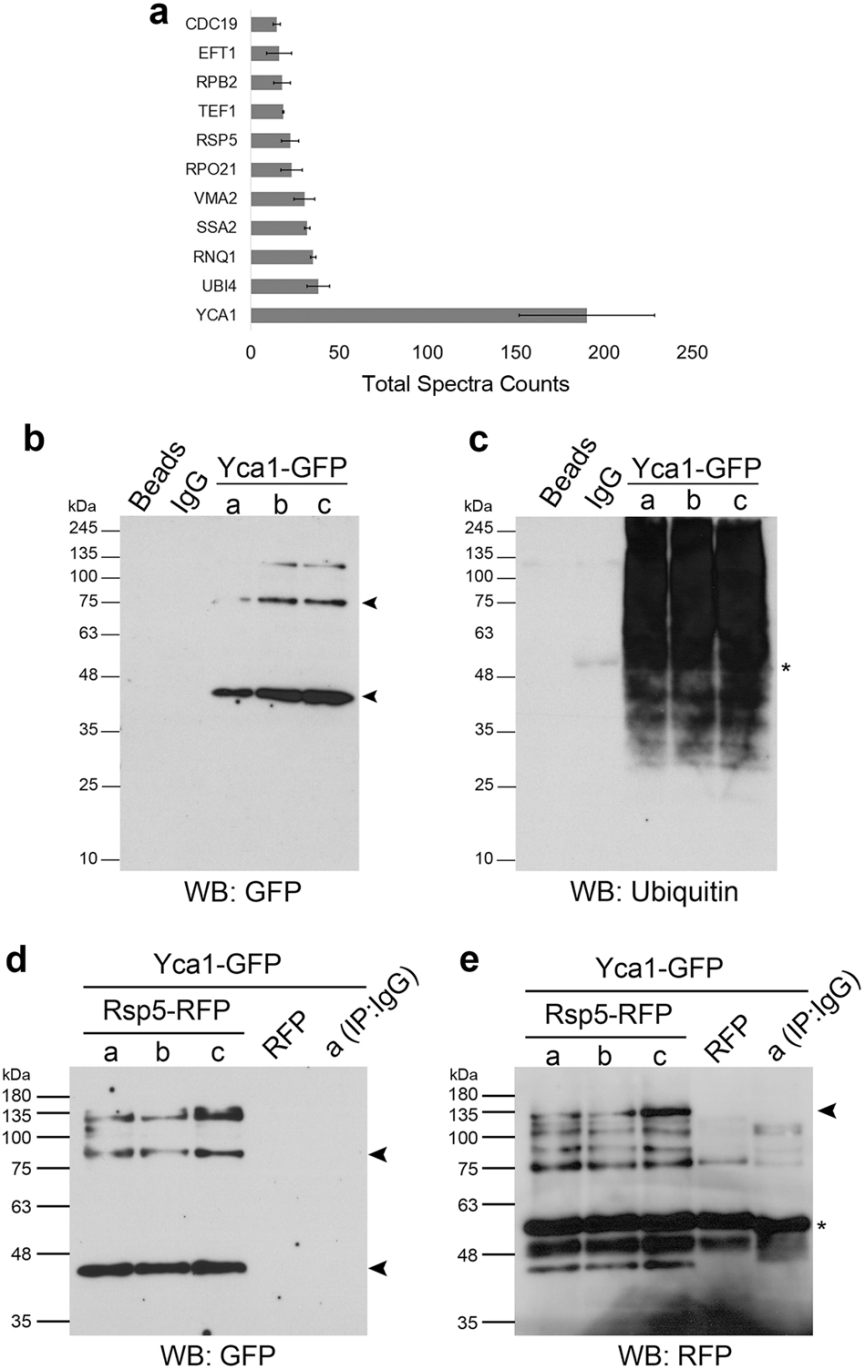
Yca1 interacts with components of the ubiquitin pathway. **a** LC-MS/MS analysis of Yca1-RFP pulldown depicted ubiquitin (Ubi4) and the E3 ligase Rsp5 in high abundance (Total Spectra Counts). Data represented as mean ± SEM. *n* = 3. **b** Yca1-GFP cells were used to isolate ubiquitinated proteins which was further probed with anti-GFP antibody to detect the presence of Yca1-GFP. The arrows highlight the full length and processed forms of Yca1-GFP. Predicted size of full length Yca1-GFP is 76 kDa and processed form in 37 kDa. Protein G beads and anti-IgG antibody conjugated beads were used as controls. Lowercase letters denote independent replicates. *n* = 3. **c** Extracts in **b** were probed with anti-ubiquitin antibody to detect the presence of the bait. The asterisk (*) highlights the presence of IgG. **d** Rsp5-RFP was expressed from a plasmid in the Yca1-GFP strain and assessed for interaction between Yca1 and Rsp5 via anti-RFP immunoprecipitation followed by anti-GFP immunoblotting. Lowercase letters denote replicate samples. The arrows highlight the full length and processed forms of Yca1-GFP. The empty vector transformed Yca1-GFP cells (RFP) was used as an interaction control. Anti-IgG antibody conjugated beads were used to probe replicate ‘a’ as an additional control. *n* = 3. **e** Extracts in **d** were probed with anti-RFP antibody to detect the presence of the bait Rsp5-RFP (highlighted with an arrow). The estimated size of Rsp5-RFP is 119 kDa. The asterisk highlights the presence of IgG

To identify components that may regulate Yca1 function, we conducted immunoprecipitation analyses coupled to proteomic identification. Our experimental model consisted of the Yca1 sequence fused to the mCherry tag (RFP), which was expressed from a plasmid under the control of the Adh1 promoter in the ∆*yca1* background^7^. This approach is an advance over our prior experimental model, as the constitutive expression of Yca1 in the current study is conducive for increased capture of protein interactions across all stoichiometries, which is an advance over our prior experimental model of defining Yca1 protein partners^7,21^. Proteins interacting with Yca1 were isolated by co-immunoprecipitation against the tag and identified using LC-MS/MS. We identified 145 proteins of high confidence, that were associated with Yca1-RFP from three independent experiments (Supplementary Table S1). We further sorted this list based on protein abundance (Total Spectra Counts; Fig. 1a, Supplementary Table S1) and noted that ubiquitin (Ubi4) was one of the most abundant proteins identified across the dataset. We also observed that the E3 ligase Rsp5 was similarly well represented as an Yca1-interacting protein.

The presence of these ubiquitin-related proteins suggested potential collaboration between Yca1 and the well characterized ubiquitin proteasome system. To verify these predicted interactions, we performed reverse immunoprecipitations coupled with immunoblotting detection. First, we isolated total ubiquitinated proteins from the Yca1-GFP (endogenously GFP tagged Yca1) strain via a pan ubiquitin immunoprecipitation and observed that Yca1 was present within this pool of ubiquitinated proteins (Fig. 1b, c). A similar approach was taken to verify the interaction between Yca1 and Rsp5; Yca1-GFP cells expressing the Rsp5-RFP plasmid were used to isolate proteins interacting with Rsp5. Upon anti-GFP immunoblotting, we observed that Yca1-GFP was present within the Rsp5 interactome (Fig. 1d, e). Together, these results confirmed the observations with the MS data.

### Yca1 is post-translationally modified by ubiquitin and the ubiquitin ligase Rsp5

The interaction between Yca1, Rsp5, and ubiquitin suggests that Yca1 may be post-translationally modified by Rsp5, which led us to identify whether specific sites on Yca1 were modified by ubiquitin. Using the peptide data from the MS analysis, the Scaffold PTM software output depicted ubiquitin tags on five sites for the Yca1 protein with high probability. Residues K163 K158, K170, K352, K355, and K384 on Yca1 were modified with the GG tag which results from the tryptic digest of ubiquitin during sample preparation (Table 1)^22^. Moreover, three of these sites K170, K352, and K355 were previously reported^23^, affirming the validity of our observations. Of note, the early studies that characterized Yca1 identified the gene as giving rise to a 454 amino acid long protein containing the catalytic cysteine at position 297^1,24^. However, additional isoforms of Yca1 have also been identified, which has led to alternate numbering of critical residues^25,26^. Our numbering of residues corresponds to the longer isoform that was first identified and physiologically characterized^5,7,21,24^, and is continued herein.

**Table 1:** Post-translational modification sites on Yca1

Site	Peptide sequence	Modification	Localization (%)	Ascore
K158	kALIIGINYIGSK	K1 GlyGly	100	110.54
K170	ALIIGINYIGSkNQLR	K12 GlyGly	100	1000
K352	AALIGSLGSIFkTVK	K12 GlyGly	100	1000
K355	TVkGGMGNNVDR	K3 LeuArg GlyGly	100	1000
K384	FSAADVVMLSGSk	K13 GlyGly	100	1000
S346	AALIGsLGSIFK	S6 Phospho	100	29.64

LC-MS/MS data from the FL-RFP immunoprecipitation was scanned for peptides containing modifications corresponding to the ‘LeuArgGlyGly’ (LRGG) and ‘GlyGly’ (GG) post-tryptic digest remnant of ubiquitin as well as for Ser/Thr phosphorylation modifications. The various modified sites on Yca1 along with the peptide sequence and modification observed are listed above. The lowercase letter within the peptide sequence indicates the position of the amino acid residue where the modification was observed. The localization probability and the Ascore for each site reflects the positional accuracy of the modification

**Fig. 2:**
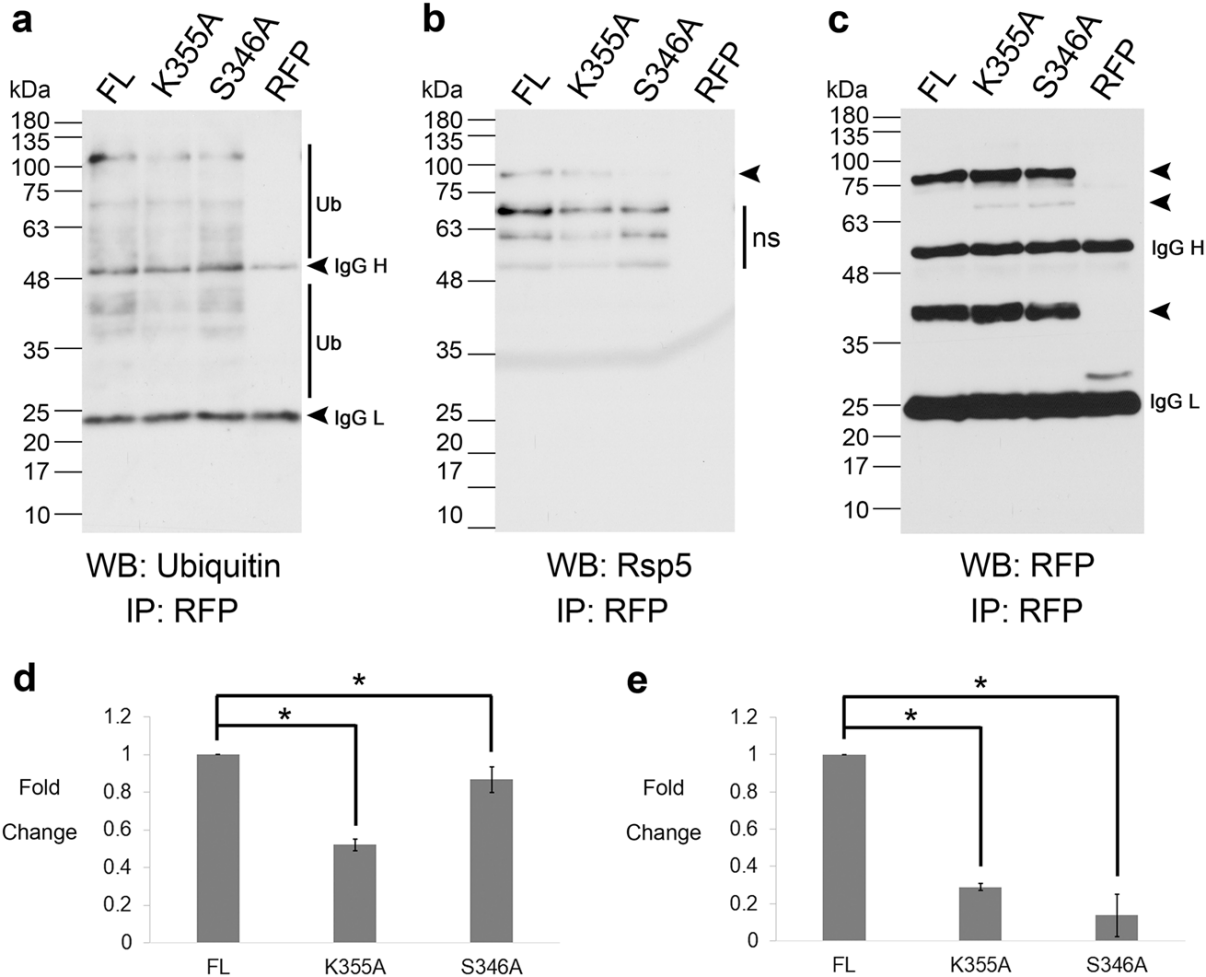
Ubiquitination at K355 is mediated by S346 dependent on the interaction with Rsp5. **a** Proteins interacting with FL, K355A, S346A and RFP (vector control) were isolated and probed for presence of ubiquitin and ubiquitin conjugate material with anti-ubiquitin antibody. *n* = 3. Ub – highlights ubiquitin bands and IgG H/L highlights immunoglobin heavy and light chains. **b** Extracts from **a** were probed for the presence of Rsp5 using anti-Rsp5 antibody. The arrow highlights the position of Rsp5. The expected size of Rsp5 is 92 kDa. ‘ns’ identifies non-specific bands. *n* = 3. **c** Extracts from **a** were probed for the presence of the respective fusion proteins via anti-RFP immunoblotting. The arrows highlight the full length and processed from Yca1-RFP. The expected size of the full length fusion proteins is 76 kDa. The expected sizes of the processed forms of the fusion proteins are 63 kDa and 37 kDa. IgG H/L highlights immunoglobin heavy and light chains. *n* = 3. A shorter exposure for this blot is depicted in Supplementary Fig. S1c, which was used for densitometry and normalization. **d** Graph depicting the level of interaction between FL, K355A and S346A and ubiquitin and ubiquitinated material as assessed via densitometry, normalized to the amount of bait (individual RFP fusion) recovered in **c** (sum of bands indicated with arrows). *n* = 3. Data represented as mean ± SEM. *p value <0.05 using ANOVA and Dunnett *t* test. **e** Graph depicting the level of interaction between FL, K355A and S346A and Rsp5 as assessed via densitometry, normalized to the bait captured in **c**. *n* = 3. Data represented as mean ± SEM. *p value < 0.05 using ANOVA and Dunnett *t* test

Next, we tested the relevance of these residues as bona fide ubiquitin modification sites. We mutated each respective lysine (K) residue to an alanine (A) in the Yca1 coding sequence and expressed them as mCherry fusions from plasmids in the ∆*yca1* background strain. Using immunoprecipitations coupled with immunoblotting, we observed that wildtype full-length Yca1-RFP (hereafter referred to as FL) associated with the prototypical ubiquitin banding pattern (Fig. 2a and Supplementary Fig. S1a, b). Densitometry analyses revealed a significant reduction in the interaction between these ubiquitin conjugates and the K355A mutant when compared to FL (*p* *<* 0.05; Fig. 2d). For the remaining four Yca1 ubiquitin mutants, the interaction with ubiquitin and ubiquitinated material was unchanged (Supplementary Fig. S1a, b). Thus, while multiple sites on Yca1 are modified with ubiquitin, the K355 residue alone was critical for establishing the Yca1 interaction with ubiquitin and ubiquitin conjugated material.

### Phosphorylation of Yca1 mediates the interaction with Rsp5

The analysis of post translational modifications (PTM) on Yca1 also led to the identification of a regulatory phosphorylation event. The serine residue at position 346 (S346), which is adjacent to the K352 and K355 ubiquitin modification sites, was observed to be phosphorylated within our MS/MS data (Table 1). Interestingly, Rsp5 belongs to the NEDD4 family of E3 ligases and the WW domains of these ligases have been shown to recognize and bind substrates in a phosphoserine dependent manner^14,27,28^. To assess whether the phosphorylation competent S346 residue may be involved in the interaction between Yca1 and Rsp5, we expressed the S346A Yca1 mutant from a plasmid as an RFP fusion in the ∆*yca1* strain. Immunoprecipitation followed by anti-Rsp5 immunoblotting showed that the association between Rsp5 and the S346A mutant was significantly reduced and this association was also inhibited in the K355A mutant (*p* *<* 0.05; Fig. 2b–e). We reasoned that if Rsp5 is the operative E3 ligase that ubiquitinates Yca1, then disturbing the interaction between Rsp5 and Yca1 should lead to a significant reduction in ubiquitin tagging of the protease. We tested this hypothesis by monitoring the interaction between the S346A mutant and ubiquitinated material and observed a statistically significant alteration (Fig. 2a–d). The level of bait (RFP) recovered from the immunoprecipitation experiments (Fig. 2c) was used for normalization to accurately assess for interaction with ubiquitin and Rsp5. Taken together, these results imply that interaction and ubiquitination of Yca1 at the K355 residue by Rsp5 is dependent on the presence of the upstream phospho-competent S346 residue, i.e. phosphorylation establishes ubiquitination competence for Yca1.

### K355A and S346A mutants affect Yca1’s proteostasis functions

**Fig. 3:**
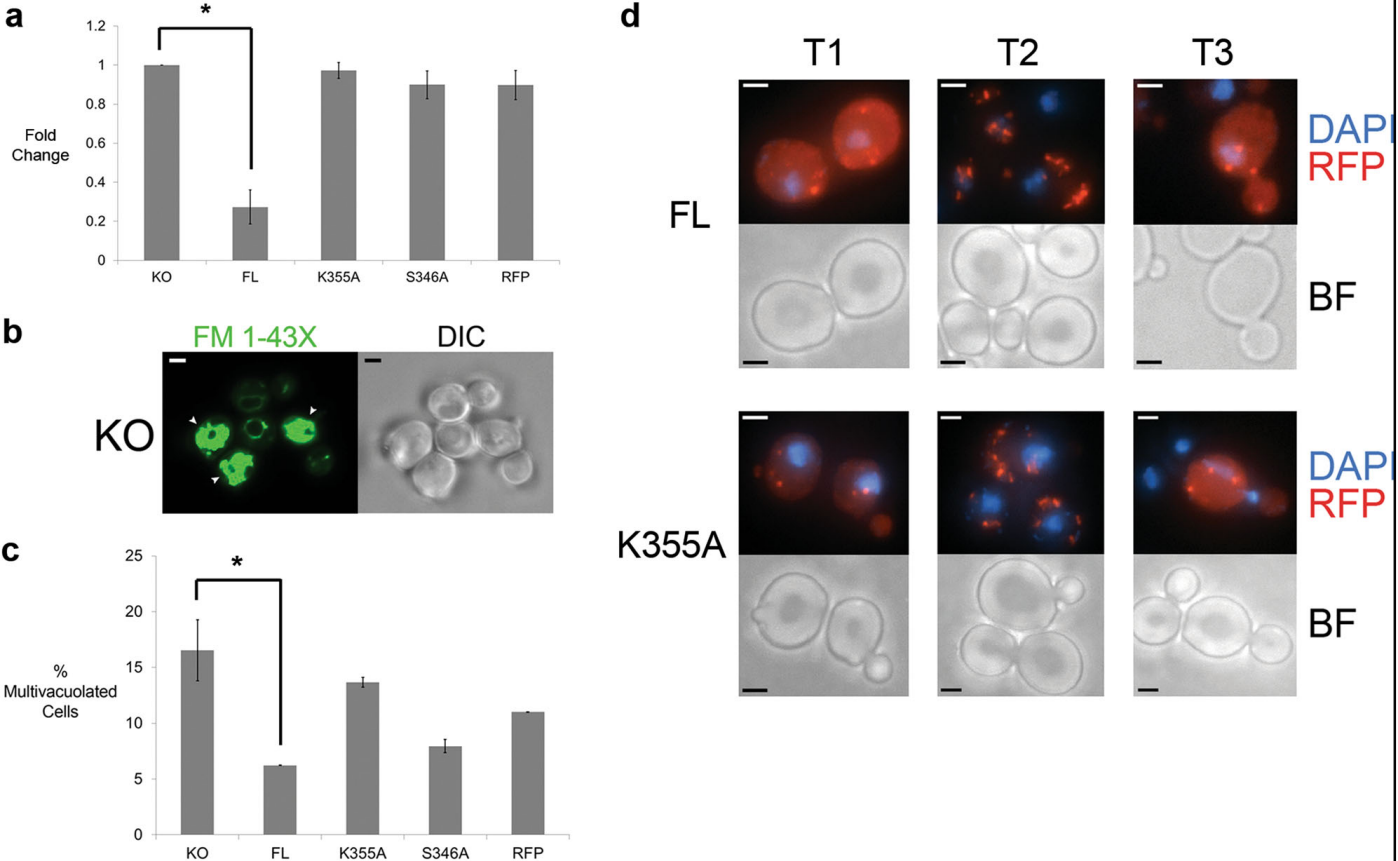
Ubiquitination of Yca1 is required for direct and indirect proteostasis control. **a** The level of cellular insoluble protein was quantified and normalized to the Yca1 knockout (KO) strain. The graph represents mean±SEM. *n* = 3 independent experiments. *p value<0.05 using ANOVA and Dunnett *t* test. **b** FM 1-43X stained KO cells depicting vacuole morphology. The white arrows highlight cells that are multivacuolated. The scale bar denotes 2 µm. **c** Representation of the proportion of multivacuolated cells in Yca1 knockout (KO), FL, K355A, S346A and vector control (RFP) cells. The data is represented as mean ± SEM. *n* = 3 independent experiments. *p value <0.05 using ANOVA and Dunnett *t* test. **d** FL and K355A cells were stained with DAPI to assess their localization to the nuclear quality control compartment and analyzed using fluorescence microscopy at 100× magnification. *n* = 3. The scale bar denotes 2 µm

The most prominent demonstration of Yca1′s capacity to maintain proteostasis is the accumulation of detergent (NP-40) insoluble protein when the protease is deleted or catalytically inhibited^7^. As such, we measured the levels of detergent insoluble protein in K355A and S346A expressing cells in comparison to the FL expressing cells and the internal control ∆*yca1*. Photometric determination of protein concentration in the pellet fraction showed that the FL expressing cells had a reduced level of insoluble material compared to ∆*yca1* (KO) strain (Fig. 3a), yet cells expressing the K355A and S346A mutants displayed increased levels of detergent insoluble protein, similar to the ∆*yca1* (KO) strain (*p* *<* 0.05). In addition to the elevation of insoluble protein content in ∆*yca1* cells, prior studies confirmed increased expression of vacuolar peptidases and vacuole structures in the ∆*yca1* strain^7^. We assessed this autophagic response in cells expressing the K355A and S346A mutants using the lipophilic dye FM^®^ 1–43 × (Fig. 3b) and noted that both the K355A and the S346A mutant expressing cells were similar to the ∆*yca1* strain with a larger number of multivacuolated structures compared to the FL expressing cells (*p* *<* 0.05; Fig. 3c).

### Impact of ubiquitination on Yca1 localization

One consequence of protein ubiquitination is altered subcellular localization of the targeted protein^11^. Pioneering studies in yeast have demonstrated that polyubiquitination of the prion Rnq1 favors its segregation into the juxtanuclear quality control compartment (JUNQ) which is tethered to the nucleus^29,30^. Prion proteins are structurally amyloid-like, which resemble aggregates associated with protein misfolding disorders such as Parkinson’s disease, Alzheimer’s disease, and Amyotrophic lateral sclerosis (ALS). Hence prion containing proteins form excellent candidates to study the aggregation and disaggregation/removal phenomena. Yca1 also localizes to the JUNQ, however, the trafficking details have yet to be investigated^8^. To determine whether ubiquitination influenced Yca1 localization, ∆*yca1* cells expressing the FL and K355A fusions were stained with DAPI under three different conditions: normal growth conditions (30 °C; T1), heat stress conditions (42 °C for 1 h; T2), and post-stress recovery conditions (45 mins at 30 °C after heat stress; T3) and analyzed via fluorescence microscopy. The inclusion of heat stress was based on the principle that under such conditions misfolded protein generation exceeds the degradation capability of the cell leading to the generation of defined quality control compartments such as the JUNQ^31^. Additionally, ubiquitin expression has also been observed to be elevated during such stresses^32^. Thus, analyzing the trafficking of FL and K355A under such conditions would be an essential aspect of cell function to query. We observed that, under normal conditions (T1) and post stress recovery conditions (T3), FL and K355A distribution was diffuse, with the formation of a few district foci. In contrast, during stress conditions (T2), both proteins were spatially concentrated to only form foci (Fig. 3d). We observed foci formation for both the FL and K355A at the nuclear periphery in all three conditions. Therefore, trafficking of Yca1 to the JUNQ is not dependent on the K355 residue of Yca1.

### Processing of the ubiquitin precursor protein Rps31 by Yca1

**Fig. 4:**
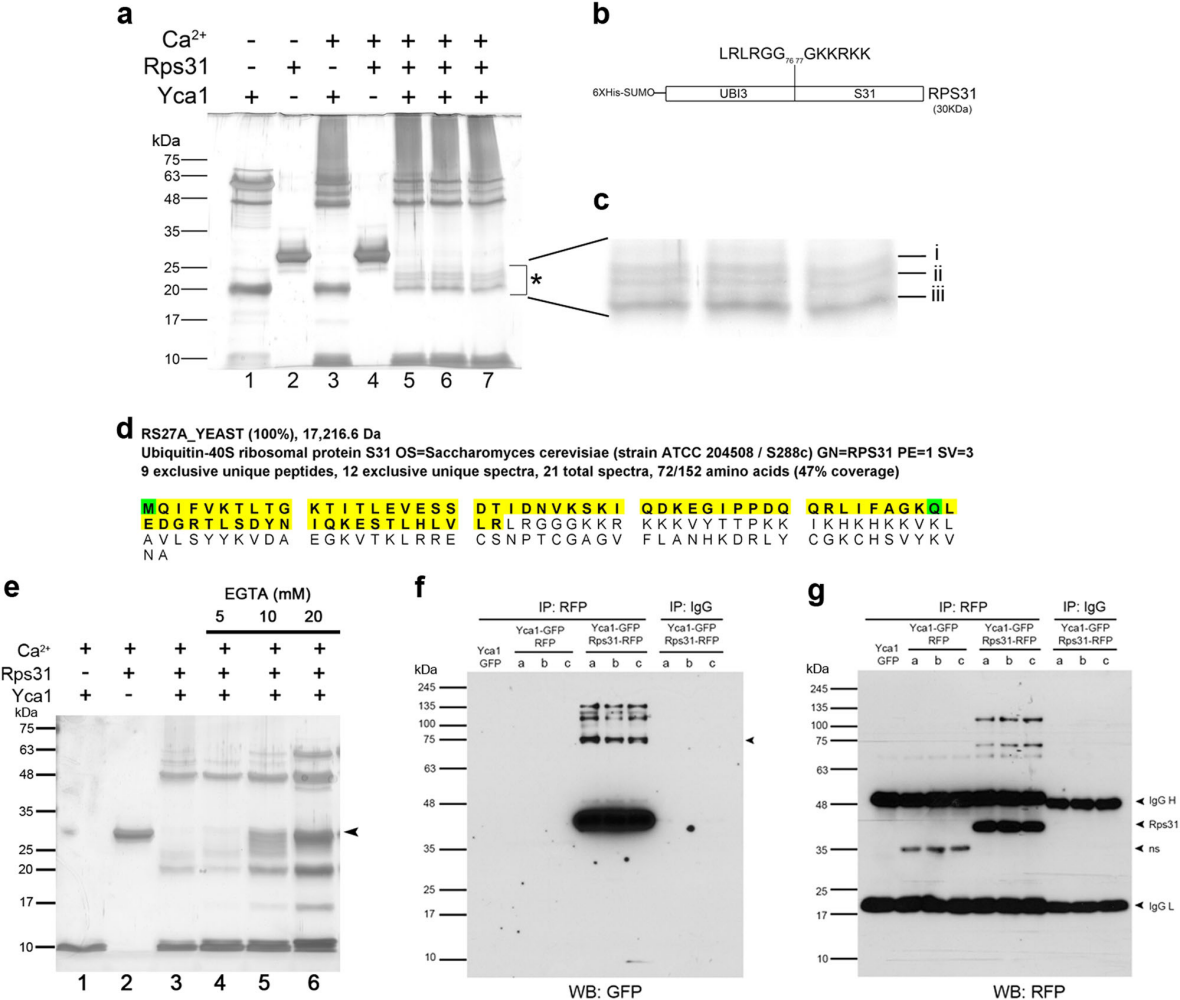
Rps31 is a substrate of Yca1. **a** Representation of the processing of Rps31 by Yca1 in the presence of calcium. *highlights the region with the cleavage products in the gel. The numbers at the bottom refer to the gel lanes. Lanes 5–7 depict experimental replicates. The estimated size of 6XHIS-SUMO-Yca1 is 64 kDa and 6XHIS-SUMO-Rps31 is 30 kDa. **b** Schematic of the Rps31 depicting the 6XHIS-SUMO purification tag, the ubiquitin (UBI3) and the ribosomal protein (S31) domains within. The sequence above highlights the junction between the C-terminal of ubiquitin and downstream lysine (K) rich residues of the N-terminal of S31. **c** Magnification of cleavage products highlighted in **a**. The lowercase letters denote the bands which were subjected to LC-MS/MS analysis. *n* = 2 independent analyses. **d** Depiction of the protein coverage of Rps31 from the LC-MS analysis of in vitro cleavage assay fragments products (**c**) as shown by Scaffold. The highlighted sequence (yellow) depicts the regions within Rps31 which were detected within the three cleavage fragments via LC-MS. The residues highlighted in green indicated modified residues during sample prep. **e** in vitro cleavage assay depicting the inhibition of Rps31 cleavage by Yca1 upon increasing concentration of EGTA (indicated above). The arrow highlights the full-length 6XHIS-SUMO-Rps31 substrate. The numbers at the bottom identify the different gel lanes. Lanes 4–6 are experimental replicates. **f** Yca1-GFP cells were transformed with the Rps31-RFP plasmid to test for interaction between Yca1 and Rps31 via anti-RFP immunoprecipitation followed by anti-GFP immunoblotting. IgG beads and empty vector (RFP) were used as controls. The arrow highlights the full-length Yca1-GFP (expected size is 76 kDa). The lowercase letters denote independent experimental replicates. **g** Extracts in **f** were probed with anti-RFP to detect the different baits used in the immunoprecipitation (highlighted with arrows). The expected size of Rps31-RFP is 43 kDa. The lowercase letters denote independent experimental replicates. *n* = 3

Further examination of the MS data from the FL-RFP immunoprecipitation suggested that the interaction with ubiquitin (Ubi4) also clustered with ubiquitin precursor ribosomal fusions. These precursor proteins undergo cleavage to generate free monomeric ubiquitin^33,34^. Examination of the protein coverage provided in the MS data identified peptides only encompassing the ubiquitin domain of the precursor protein Rps31, suggesting an enzyme–substrate relationship between Yca1 and this protein. To address whether Yca1 regulates *de novo* ubiquitin synthesis, we conducted cleavage assays in vitro, using the Rps31 ubiquitin precursor protein as a substrate. Recombinant Yca1 and Rps31 were commercially generated and assayed using calcium as an enhancer for metacaspase activity^26,35^. We observed that the full-length Rps31 substrate was completely processed in samples containing active Yca1 (lanes 5–7; Fig. 4a), suggesting that the observed fragments were the result of metacaspase directed cleavage. Subsequent LC-MS analysis of these fragments (Fig. 4c), confirmed a 100% match to Rps31 peptides and the Smt3 protein from the tag (6XHIS-SUMO; Fig. 4b). Examination of protein coverage using the Scaffold software confirmed that these bands contained the N-terminal ubiquitin domain (Ubi3) of Rps31 but not the C-terminal S31 ribosomal protein (Fig. 4d). Thus, the ubiquitin precursor protein Rps31 is amenable to cleavage by Yca1, leading to the separation of the ubiquitin moiety from the precursor. In addition, it is important to note that the cleavage ability of Yca1 has been shown to strongly depend on the presence of calcium^26^. As such, we examined calcium dependency in our cleavage assay by using various concentrations of EGTA to chelate Ca^2+^ ions. We observed an inhibition in the cleavage of the full-length 6XHIS-SUMO-Rps31 substrate which correlated with increasing EGTA concentration (Fig. 4e), which suggests that Ca^2+^ can regulate the activity of Yca1.

**Fig. 5:**
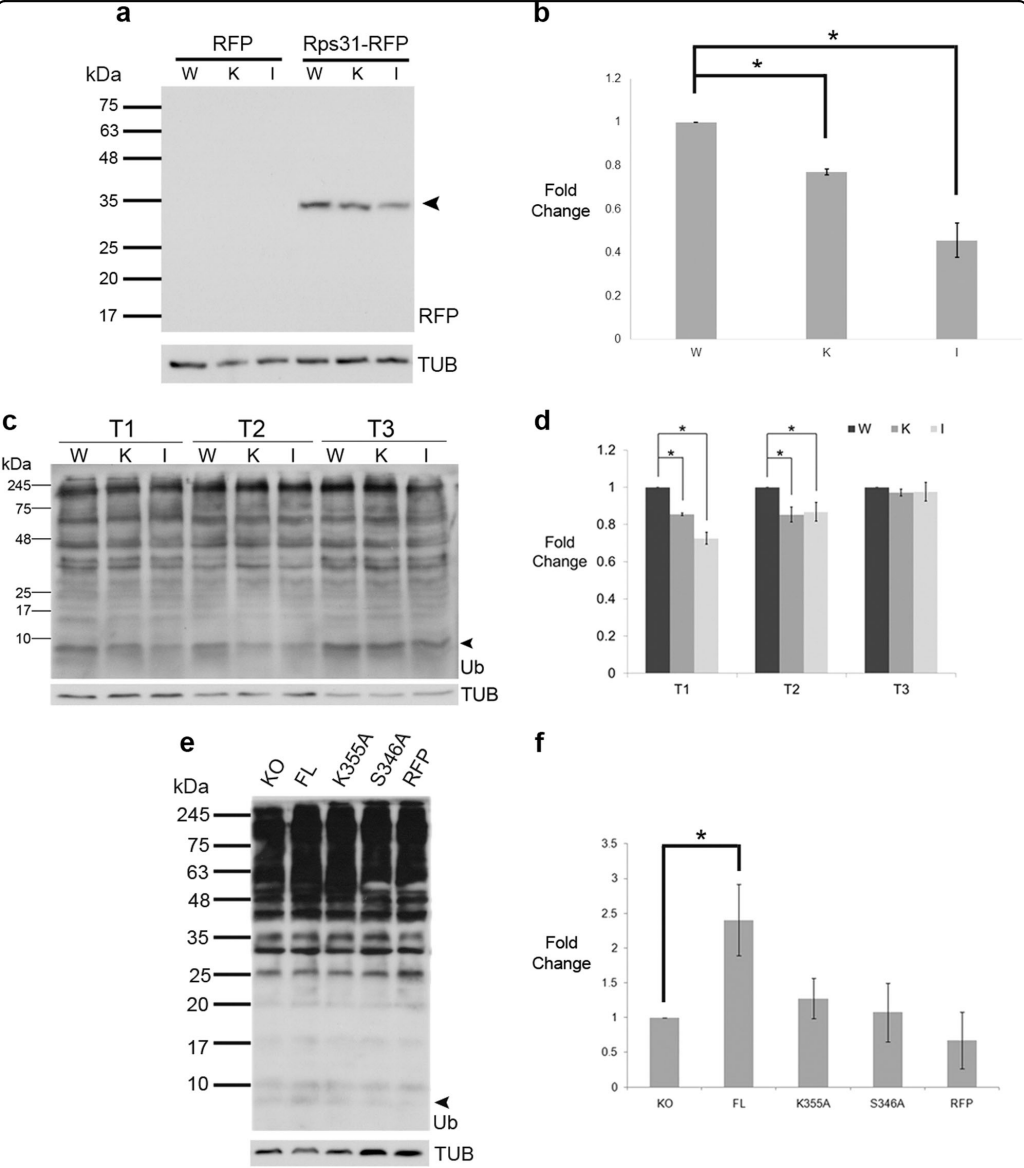
Yca1 is involved in the regulation of cellular monomeric ubiquitin levels. **a** Wildtype BY4741 (W), Yca1 knockout (K), and the catalytically inactive C297A mutant (I) cells were transformed with the Rps31-RFP plasmid or empty vector (RFP) and assessed for the expression of the fusion protein via anti-RFP immunoblotting. Tubulin was used as loading control. *n* = 3. **b** Graph depicting the fold change in detection of the Rps31-RFP cleavage product between the three cell types normalized to the wildtype (WT). The data is represented as mean ± SEM. *p value < 0.05 using ANOVA and Dunnett *t* test. **c** The ubiquitin profile for wildtype (W), ∆*yca1* (K), and inactive Yca1 mutant C297A (I) from normal (T1), heat stress (T2), and post stress recovery (T3) conditions. The arrow highlights low molecular weight ubiquitin quantified in **d**. *n* = 4 independent experiments. **d** Quantification of ubiquitin band highlighted in **c**, normalized to the wildtype. The data is represented as mean ± SEM. *p value < 0.05 using ANOVA and Dunnett *t* test. **e** The ubiquitin profile for ∆*yca1* (KO), FL, K355A, S346A, and RFP under normal conditions. The arrow highlights low molecular weight ubiquitin quantified in **f**. Tubulin was used as a loading control. *n* = 3. **f** Quantification of the band highlighted in **e**, normalized to the Yca1 knockout (KO). The data is represented as mean ± SEM. *p value < 0.05 using ANOVA and Dunnett *t* test

To confirm whether Rps31 is a substrate for Yca1 in vivo, we measured the interaction between Yca1 and Rps31 by immunoprecipitation methods. Using the same model described for the confirmation of the Rsp5-Yca1 interaction, we transformed endogenously GFP tagged Yca1 cells with the Rps31-RFP expression plasmid. The interaction between Yca1-GFP and Rps31-RFP was confirmed via anti-GFP immunoblotting of the extracts from the anti-RFP pulldown (Fig. 4f, g). Next, we transformed wildtype (BY4741; W), Yca1 knockout (K), and catalytically inactivated C297A mutant (I) cell lines with plasmid expressing the Rps31-RFP fusion protein and probed for its expression in the three backgrounds. We observed a band with a size corresponding to the size of the cleaved Rps31-RFP (35 kDa; Fig. 5a). Densitometry analysis of this band between the three backgrounds depicted that the loss (K; ∆*yca1*) or catalytic inactivation of Yca1 (I; C297A) correlates with reduced amount of Rps31-RFP cleavage product (*p* *<* 0.05; Fig. 5b).

### Loss of Yca1 or its activity reduces low molecular weight ubiquitin in vivo

If Yca1 acts to generate monomers of ubiquitin, then loss of this protease would be expected to reduce the level of free ubiquitin available for proteome regulation. We examined the ubiquitin profile via immunoblotting on extracts prepared from the wildtype BY4741, ∆*yca1* and the catalytically inactive Yca1 (C297A) strain^5^. Under normal growth conditions (T1), the level of monomeric ubiquitin (indicated with an arrow; Fig. 5c) was observed to be reduced in both the ∆*yca1* cells (K) and C297A (I) cells (*p* *<* 0.05; Fig. 5d). Furthermore, with the advent of heat stress (T2) free monomeric ubiquitin remained at a reduced level in both the ∆*yca1* and the C297A cells (*p* *<* 0.05; Fig. 5d). However, under post-stress recovery conditions (T3), monomeric ubiquitin levels were similar in all three strains, suggesting an alternate source of ubiquitin generation at this stage of adaptation.

Detection of protein lysates on a 12% gel (Fig. 5c) indicated that the migrating mono-UB species may consist of a doublet and /or be obscured by other low migrating forms of conjugated UB species. To further resolve the identity of the UB forms, we conducted subsequent experiments using a 15% gel. Specifically, we assessed whether the K355 or the S346 residues affected Yca1’s role in regulating ubiquitin levels in vivo and we observed that FL Yca1 cells had a greater level of the low molecular weight ubiquitin (arrow) compared to the Yca1 knockout cells (KO) (*p* < 0.05; Fig. 5e, f). Furthermore, the K355A and the S346A mutants possessed this band at a similar level to that observed for the KO strain (Fig. 5e, f), suggesting that these residues influence the role that Yca1 may have in regulating mono-ubiquitin levels.

## Discussion

Using an unbiased immunoprecipitation screen coupled to LC-MS/MS we identified a series of known and novel protein interactions for the yeast metacaspase Yca1 (Supplementary Table S1). Within this interactome, we identified both ligase and ubiquitin moieties that regulated Yca1 activity (controlling aggregation kinetics), and further confirmed the latter to be generated by Yca1 enzymatic activity. Together, these observations suggest that Yca1 controls proteostasis by managing the free ubiquitin pool, a biochemical step that (1) feeds back to tag Yca1 through direct ligase interaction which ensures the metacaspase is active, and (2) provides free ubiquitin moieties which may be deployed for general proteasome targeting and aggregate dispersal.

Further biochemical analysis identified that the modification of K355 by ubiquitin had direct impact on Yca1 function in proteostasis, i.e. regulation of insoluble protein levels and the autophagic response (Fig. 3a–c). The ability of Yca1 to regulate insoluble protein levels as well as the autophagic response suggests that this protease is involved in the crosstalk between the two degradation systems in the cell i.e. the UPS and autophagy^36^. Interestingly, this feature has been attributed to Rsp5 function in prior studies^37^. The autophagy regulation by caspase like proteases may be a conserved phenomenon, as effector caspase proteases have also been implicated as direct modifiers of the autophagic response, by targeting mitochondrial proteins that control autophagic flux^38^. Thus, Yca1-dependent autophagy regulation may be operative in yeast, alternatively the increased vacuolar density in the absence of Yca1 may simply be a secondary/compensatory response to the accumulation of protein aggregates.

Our observations also suggest that the phospho-competent S346 residue acts as an intermediary in the interaction between Yca1 and Rsp5, where the phosphorylation of the S346 residue acts as a signal to target Rsp5 for ubiquitination at K355. Our analysis did not identify the kinase involved in phosphorylating S346, however, we identified several candidates within the Yca1 interactome, including the Ser/Thr kinase Ypk1 (Supplementary Table S1). Moreover, an independent investigation using the PhosphoMotif Finder tool (www.hprd.org) identified the S346 residue in the ‘SLGS’ sequence (residues 346–349) as a substrate recognition motif for the kinase CK1, whose substrates have been shown to be protected from caspase cleavage^39^. For example, phosphorylation of Pax7 by CK2 was observed to protect Pax7 from caspase 3 cleavage, which was required for the self-renewal of satellite cells and prevention of the myogenic differentiation program^40^. Thus, it may be reasonable to speculate that the phosphorylation of S346 in Yca1 may also influence processing of Yca1 at the K355 residue.

Ubiquitination of various caspase proteins has also been correlated to their proteolytic activity. Cullin3-mediated K63 ubiquitination of caspase 8 leads to p62-mediated caspase 8 activation^41^. Alternatively, HECTD3-mediated K63 ubiquitination of caspase 8 has been observed to be inhibitory as it limited its recruitment to the death-induced signaling complex (DISC)^42^. Thus, the ubiquitination of metacaspases may serve an analogous function depending on the E3 ligase that mediates the process. The structural analysis of Yca1 suggested that the K355 residue along with the adjacent K352, and upstream R93 and K107 residues were processing sites, although this hypothesis was not further investigated^26^. Our data suggests that two of these processing sites are modified by ubiquitin (Table 1), which in turn may modulate Yca1 activity.

Yca1 has been observed to localize to distinct quality control compartments such as the JUNQ in aging yeast, which is at a time when proteostasis mechanisms are in a steady decline^8^. The ubiquitination of various misfolded protein substrates has been suggested to be a limited sorting factor for the JUNQ^29,43^. We assessed the nuclear localization of the K355A mutant to further verify if such a modification could also recruit non-substrate proteins to these quality control compartments. Our analysis suggests that modification of the K355 residue does not serve as a relocation signal. Nonetheless, analysis of the four additional ubiquitin modification sites may be more informative regarding Yca1 ubiquitination and sorting to subcellular compartments.

The observation that the ubiquitin precursor protein Rps31 was processed by Yca1 to liberate the N-terminal ubiquitin moiety in vitro (Fig. 4a–d) and cleave the Rps31-RFP reporter in vivo (Fig. 5a, b), suggests a novel role for Yca1 in de novo ubiquitin synthesis. Indeed, this hypothesis is consistent with the reduced levels of low molecular weight ubiquitin observed upon the loss of Yca1 or its catalytic activity in vivo (Fig. 5c, d). Additionally, the reduced levels of low molecular weight ubiquitin in K355A and S346A cells (Fig. 5e, f), suggests that these residues may also influence Yca1’s role in ubiquitin synthesis. In mammals, deubiquitinating enzymes (DUBs) have been shown to process precursor ubiquitin ribosomal fusions^34^. However, no reports detailing the biochemical testing of precursor ubiquitin ribosomal fusion cleavage events in yeast have been described. Our examination here highlights the first evidence of a metacaspase directed cleavage of ubiquitin precursors, which suggests that Yca1 may act as a de novo DUB enzyme. Based on the R/K substrate specificity of metacaspases^26,44,45^, the Rps31 precursor represents a strong candidate for cleavage as it contains a series of K residues downstream of the C-terminal G76 residue of the ubiquitin domain (Fig. 4b). These downstream residues contain a motif that was observed to be frequently cleaved by the plant metacaspase AtMC9^46^. Analysis of the Rps31 domains showed that the ubiquitin moiety is required for the assembly of the S31 protein to form functional 40 S ribosomal subunits as the loss of this moiety was observed to be lethal^13^. As such, we speculate that the cleavage event by Yca1 would ensue after the incorporation of the S31 domain into the 40 S subunit. In addition, the presence of Yca1 in ubiquitin-rich regions such as the JUNQ is suggestive of a role for Yca1 in generating ubiquitin monomers, a mechanism that would ensure a healthy pool of free ubiquitin for protein sorting and processing. Interestingly, Yca1 and the DUB ATXN3, are both cysteine proteases and possess similar structural features such a *N*-terminal polyglutamine-rich region^47^. Of note, ATXN3 belongs to the Machado-Joseph Disease (MJD) family of DUBs, although homologs of this family have yet to be discovered in yeast^15^.

Whether Yca1 can cleave other ubiquitin precursors and polyubiquitin chains remains to be examined. Specifically, DUBs have been shown to be inhibited by the sulfhydryl alkylating agent *N*-ethylmaleimide (NEM), particularly by modifying the reactive cysteines within these enzymes^48,49^. Initially, we had also conducted immunoprecipitation for FL to identify its interactors with NEM to test ubiquitin targeting of Yca1. Accordingly, pretreating recombinant Yca1 with NEM inhibited its processing and cleavage of the Rps31 substrate (Supplementary Fig. S2a). Furthermore, the MS data suggested that the catalytic cysteine C297 was also modified by NEM (Supplementary Fig. S2b, c), suggesting that NEM based experiments do not solely monitor ubiquitin related alterations in enzymatic activity. Indeed, we also identified a second cysteine residue that was modified by NEM, C176, suggesting that Yca1 may be similar to other type I metacaspases, in possessing two catalytic cysteine residues^50,51^.

**Fig. 6:**
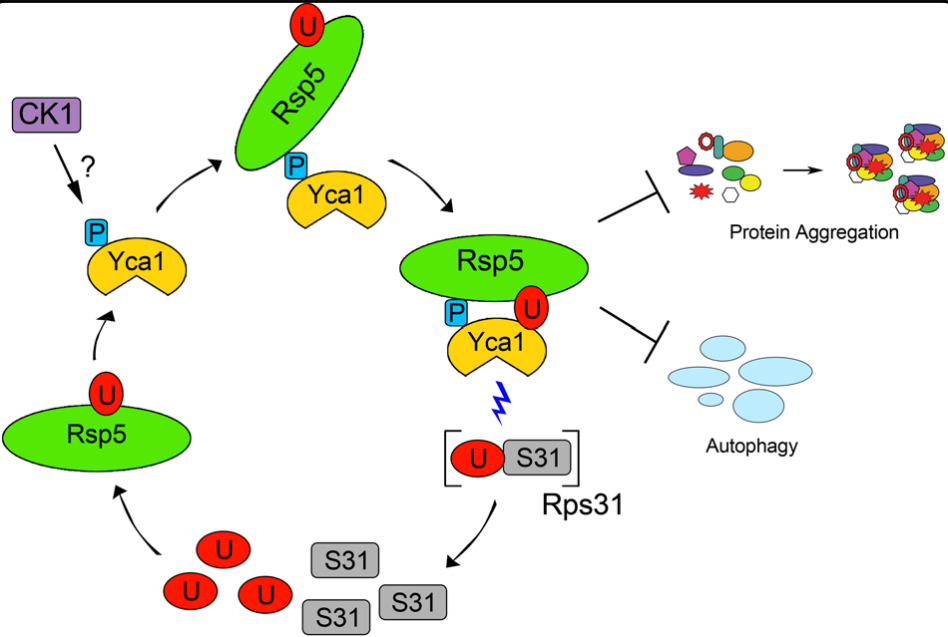
Yca1 maintains proteostasis through direct participation in the ubiquitin cycle. Phosphorylation of the S346 residue (P) on Yca1 by candidate kinases such as casein kinase 1 (CK1), is recognized by Rsp5, which leads to ubiquitination (U) at K355. In turn, these modifications influence Yca1’s ability to limit protein aggregation and autophagy as well as target and liberate free ubiquitin from precursor proteins (Rps31). Once liberated, the free ubiquitin may cycle back to maintain Yca1 activity or be used for modifying other proteins to maintain proteostasis within the cell

Overall, this study provides the first evidence of direct communication between metacaspase proteolysis and the ubiquitin proteasome system (Fig. 6). Direct ubiquitination of Yca1 was observed to govern its ability to regulate proteostasis. Additionally, we noted that the metacaspase directly engaged and modified the ubiquitin system. Given the broad distribution of metacaspase enzymes, which span from fungi to plants, we suggest that the metacaspase crosstalk with the ubiquitin proteasome system is likely to be a broadly conserved biologic phenomenon.

## Materials and methods

### Strains, media and growth conditions

All *Saccharomyces cerevisiae* strains used in this study were derived from the wildtype haploid BY4741 (MATa his3∆1 leu2∆0 met15∆0 ura3∆0) strain as previously described^5,7^. All experiments were conducted in acidic YPD media as originally described^5^. Normally cells were grown to mid-logarithmic phase (T1; OD_660_ 0.5–0.6) at 30 °C. For heat shock treatment (T2), mid-logarithmic cultures were further incubated at 42 °C for 1 h before collection. For post-stress recovery growth condition (T3), cultures were incubated at 30 °C for an additional period of 45 min after the heat stress treatment. For immunoprecipitation experiments, cultures were grown to OD_660_ between 1.0 and 1.2 at 30 °C. For insoluble protein analyses, cell cultures were cultured at 30 °C and limited to a maximum culture density of 0.9 at OD_660_.

### Plasmids and cloning

The FL construct expressing the full-length Yca1 protein as a RFP fusion under the control of the ADH1 promoter^7^, was used to prepare constructs expression Yca1 mutants. The primers used to generate the respective single amino acid change in the Yca1 coding sequence are listed in Supplementary Table S2. The Rsp5-RFP and Rps31-RFP expression plasmid was generated by cloning into the pRS316 plasmid (Creative Biogene, NY, USA) with the same background as FL. All final constructs were verified by DNA sequencing analysis (StemCore Laboratories, Ottawa, Canada) prior to transformation of yeast strains using lithium acetate^7^.

### Protein extraction

Protein extracts were prepared from frozen cell pellets using various buffers. For immunoprecipitation analyses, cells were lysed in buffer A (20 mM HEPES-NaOH, pH 7.4, 2 mM MgCl_2_, 300 mM NaCl and 0.1% Tween-20). For assessing differences in ubiquitin levels, the cells were lysed in buffer B (50 mM TRIS-HCl, pH 7.4, 150 mM NaCl, 1 mM EDTA, 1% NP-40, 1% glycerol). For preparation of the insoluble protein fraction, cells were lysed in buffer C (50 mM TRIS-HCl, pH 7.4, 0.1% NP-40, 1 mM EDTA, 1% glycerol). All buffers were supplemented with protease inhibitors for lysis. Protein was extracted from cells using the glass beads method described previously^7^.

### Immunoprecipitation

Antibody conjugated magnetic beads were added to equal amounts of total protein extract for all samples. Anti-RFP mAb-magnetic beads (M165–11; MBL International, MA, USA) and Anti-Multi Ubiquitin mAb-magnetic beads (D058-9; MBL International, MA, USA) were used for isolation of interacting proteins. IgG only beads were used as controls (M075-11; MBL International, MA, USA, sc-2025; Santa Cruz Biotechnology, TX, USA). The sample was incubated overnight at 4 °C with end over end rotation. Following incubation, the beads were separated on a magnet were further washed 5 times with ice cold buffer A without protease inhibitors. Proteins were eluted in 1X laemmli buffer with or without β-mercaptoethanol for 2–3 minutes then boiled at 100 °C for 5 min. The eluted proteins were separated from the beads analyzed via SDS-PAGE and either resolved by silver nitrate staining^52^ or transferred to PVDF for immunoblotting^7^.

### Antibodies

Antibodies used in this study are described herein. To detect ubiquitin, we used the polyclonal rabbit anti-Ubiquitin antibody (ab19247; Abcam, UK). The goat anti-Rsp5 (sc-26193; Santa Cruz Biotechnology, TX, USA) polyclonal antibody was used to detect presence of Rsp5. Yca1-GFP presence was detected using the chicken polyclonal to GFP antibody (ab13970; Abcam, UK). mCherry fused proteins were detected using mouse monoclonal anti-RFP antibody (MBL International, MA, USA). This antibody does not react with GFP. Tubulin levels were detected using monoclonal rat anti-tubulin antibody (ab6160; Abcam, USA). IgG levels were detected using the goat anti-rat IgG HRP conjugate antibody (STAR113P; Bio-Rad, CA, USA). Secondary HRP conjugated antibodies used to detect the primary antibodies in this study are as follows: goat anti-mouse (170–6516), goat anti-rabbit (170–6515), rabbit anti-goat (172–1034; Bio-Rad, CA, USA), and goat anti-chicken (ab6877; Abcam, UK).

### Mass spectrometry

Proteins were digested in-gel using trypsin and chymotrypsin (Promega, USA). The resulting peptide extracts were concentrated by vacufuge (Eppendorf, Germany) and resuspended in 1% formic acid (Fisher Scientific, USA). Peptides were analyzed by liquid chromatography – tandem mass spectrometry (LC-MS/MS) on a system comprised of an UltiMate 3000 RSLC nano, LTQ Orbitrap XL hybrid mass spectrometer, and nanospray ionization source (Thermo Fisher Scientific, MA, USA). The system was controlled by Xcalibur software version 2.0.7 (Thermo Fisher Scientific, MA, USA). Peptides were loaded by autosampler onto a C18 trap column (Agilent Technologies, USA) in 3% acetonitrile, 0.1% formic acid at a flow rate of 15 µL per minute for 5 min. Peptides were eluted over a 100-min gradient of 5.6–25.6% acetonitrile at a flow rate of 250 ƞL per minute^53^, through a 10-cm analytical long column with integrated emitter tip (Picofrit PF360-75-15-N-5 from New Objective packed with Zorbax SB-C18, 5 microns from Agilent Technologies, CA, USA), and nanosprayed into the mass spectrometer. Nano-pump HPLC solvents contained 0.1% formic acid and 5% DMSO^54^. MS scans were acquired in FTMS mode at a resolution setting of 60,000. MS/MS scans were acquired in ion trap CID mode using data-dependent acquisition of the top 5 ions from each MS scan.

### Protein identification using MASCOT

For identification of proteins in the FL-RFP immunoprecipitation analyses, MASCOT software version 2.5 (Matrix Science, UK) was used to infer peptide and protein identities from the mass spectra. The observed MS/MS spectra were matched against *S. cerevisiae* sequences from SwissProt (version 2014–08) and against a database of common contaminants. Mass tolerance parameters were MS ± 10 ppm and MS/MS ± 0.6 Da. Enzyme specificity was set to ‘Trypsin’ with  ≤2 miscuts. GlyGly modification of lysine, LeuArgGlyGly modification of lysine, oxidation of methionine, protein *N*-terminal acetylation, and conversion of glutamine to pyroglutamate were allowed as variable modifications. In some searches, phosphorylation of serine or threonine, formylation of serine, dimethylation of methionine and/or oxidation of tryptophan or histidine were also used as variable modifications. Error-tolerant search was also used in some cases. The data were exported to Scaffold and Scaffold PTM software (Proteome Software, USA), for further viewing and validation.

### Vacuole staining and count

For the visualization of vacuoles, cells were stained with the FM^®^ 1-43X lipophilic dye (Thermo Fisher Scientific, MA, USA) as previously described^7^. Cells possessing >3 vacuoles/cell were counted and determined as a percentage of the total number of cells analyzed.

### Nuclear staining

Cells from mid-logarithmic cultures were collected, washed, and fixed in ice cold 70% ethanol. The cells were recollected and resuspended in 1X PBS containing 1 µg/mL of 4′,6-diamidino-2-phenylindole (DAPI) for 10 min. The cells were pelleted then resuspended in 1X PBS and spotted on microscopy slides. Stained cells were viewed under 100× magnification using the Leica DMI 6000 florescent microscope (Leica Microsystems, Germany), equipped with a Sutter DG4 light source (Sutter Instruments, CA, USA), Ludl emission filter wheel with Chroma band-pass emission filters (Ludl Electronic Products, NY, USA) and Hamamatsu Orca AG camera (Hamamatsu Photonics, Japan). Multiple images were captured using Volocity 4.3.2 Build 23 (Perkin-Elmer, MA, USA).

### Insoluble protein quantification

The insoluble protein was prepared from total protein extracts as described previously^7^. The final protein pellet was dissolved in the resolubilization buffer (6 M urea, 2% SDS, 20 mM HEPES-NaOH, 150 mM NaCl, 2 mM EDTA, 1 mM, DTT, pH 7.4; buffer modified from ref. ^55^) via vortex at 4 °C for 20 min. The dissolved insoluble protein solution was further diluted 1:3 in 1X PBS and used to determine the insoluble protein concentration spectrophotometrically.

### In vitro cleavage assay

Commercially generated recombinant 6XHis-SUMO tagged Rps31 and Yca1 in *E. coli* (ABclonal, MA, USA) were incubated together or alone in the reaction buffer (20 mM TRIS-HCl, 150 mM NaCl, pH 8.0). Calcium chloride was added to a final concentration of 15 mM for metacaspase activation^26^. Reaction was performed at 25 °C for 2 h. For assessing to dependence of Yca1 on Ca^2+^ for activity, EGTA was used to chelate Ca^2+^ at 5, 10, and 20 mM concentrations. For assessing the role of NEM in cleavage ability, Yca1 was pretreated with NEM at 5, 10, and 20 mM for 5 min at room temperature followed by the addition of Rps31 and cleavage buffer containing calcium. Reaction was carried out for 30 min at 25 °C.

### Quantification and statistical analyses

Quantification of immunoblots were performed using the ImageJ software. The concentration of insoluble protein was determined via interpolation from a standard curve generated using bovine serum albumin. The proportion of multivacuolated cells for each sample was determined via manual counting of multiple FOVs. Densitometry, insoluble protein concentration, and proportion of multivacuolated cell data was subjected to one-way analysis of variance (ANOVA) and the Dunnett *t* (2-sided) test using SPSS software. A *p* value of <0.05 was considered statistically significant and highlighted with an asterisk (*).

## Electronic supplementary material

Supplementary Information

Supplementary Table S1

## Data Availability

Datasets generated in this study has been provided as Supplementary Information.

## Electronic supplementary material

**Supplementary Information** accompanies the paper at (10.1038/s41421-018-0071-9).
