# Supplementary material for: The metacaspase Yca1 maintains proteostasis through multiple interactions with the ubiquitin system
Source: Cell Discov. 2019 Jan 22;5:6. doi: 10.1038/s41421-018-0071-9 (PMC6341073; doi:10.1038/s41421-018-0071-9)
Supplement: Supplementary file 1 — Supplementary Information [file 41421_2018_71_MOESM1_ESM.pdf]

Supplementary Information

Supplementary Fig. S1. Analysis of Yca1 mutants for ubiquitin modification sites.

a Yca1 mutants described in Table 1 were analyzed for interaction with ubiquitinated material via immunoblotting. Empty vector (RFP) and IgG were used as controls. *n*=3 independent experiments. **b** Graph represents the level of interaction observed for each Yca1 mutants and plasmid control as determined via densitometry. Data is depicted as mean ± SEM. *n*=3. **c** Lighter exposure of the anti-RFP immunoblot shown in Fig. 2c that was used for densitometry analyses. The arrows highlight the three different forms (full length 76kDa and processed 63kDa and 36 kDa) of Yca1.

Supplementary Fig. S2. N-ethylmaleimide treatment affects Yca1 activity.

a Gel depicting the inhibition of Yca1 processing upon treatment with N-ethylmaleimide (NEM). The concentration of NEM are indicated above. 'f' indicates the full length 6XHIS-SUMO-Yca1 protein and 'p1/2' indicates the processed forms of Yca1. The asterisk highlights the 6XHIS-SUMO-Rps31 substrate. The numbers on the bottom indicate gel lanes. *n*=3. **b** Protein coverage map of NEM-treated Yca1 as depicted by Scaffold PTM. The yellow highlights the regions within Yca1 that were detected by LC-MS/MS. The residues highlighted in green indicate cysteines that were observed to be modified on Yca1 by NEM. **c** Table showing the details on the modifications observed in **b** as depicted by Scaffold PTM.

Supplementary Table S1. The Yca1 Interactome.

List of proteins as interacting with the FL-RFP as depicted under the ‘quantitative value (total spectra)’ using the Scaffold software. The presence of the interacting protein in control samples (KO – Yca1 knockout and RFP – empty vector) were excluded to generate a FL-RFP only interacting protein list.

Supplementary Table S2. Primers used in this study.

Primers used to prepare RFP fusions of Yca1 mutants and the Rsp5-RFP fusion are depicted above in 5’to 3’ order. The nucleotide mismatches within the primer sequences have been underlined. The restriction site sequences within the primers are denoted with italics.

Supplementary Fig. S1

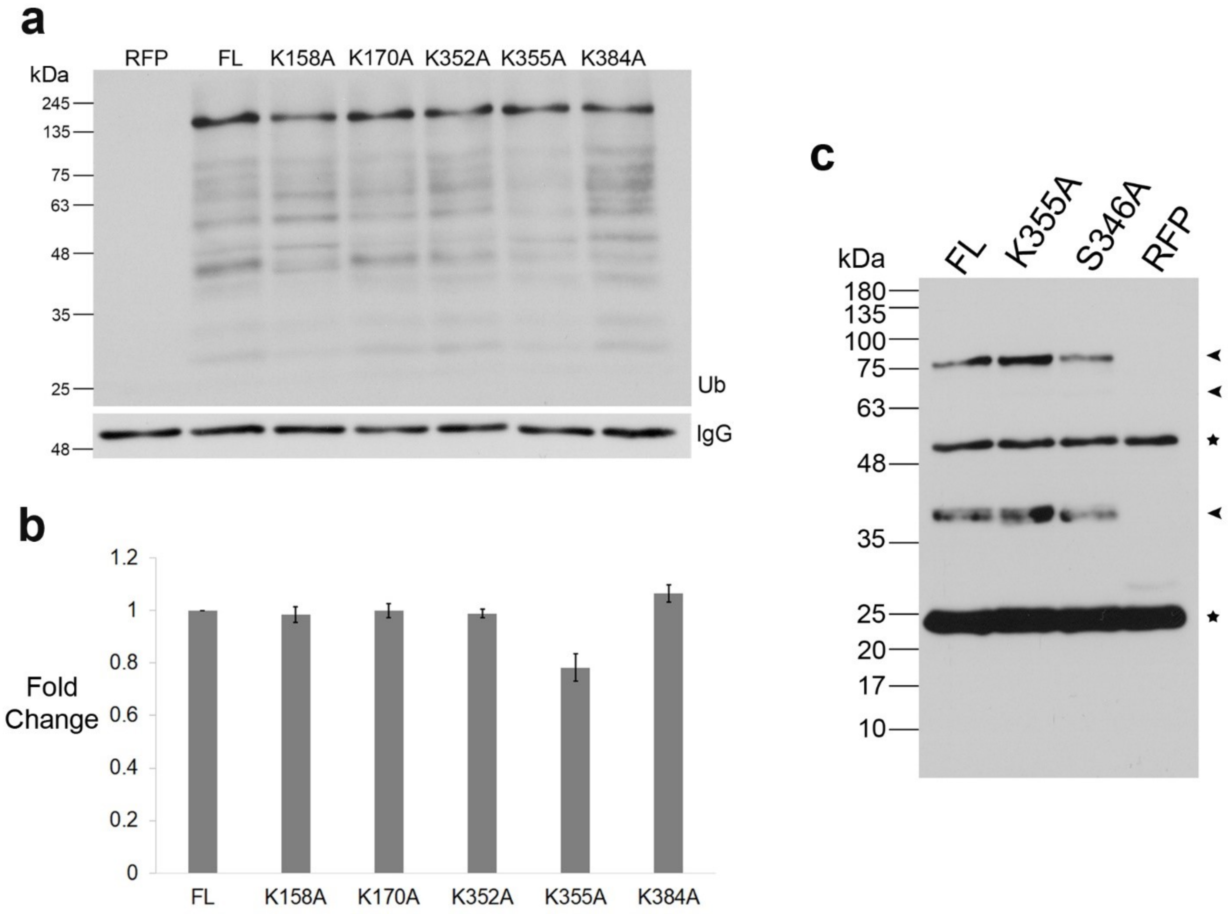

Supplementary Fig. S2

a

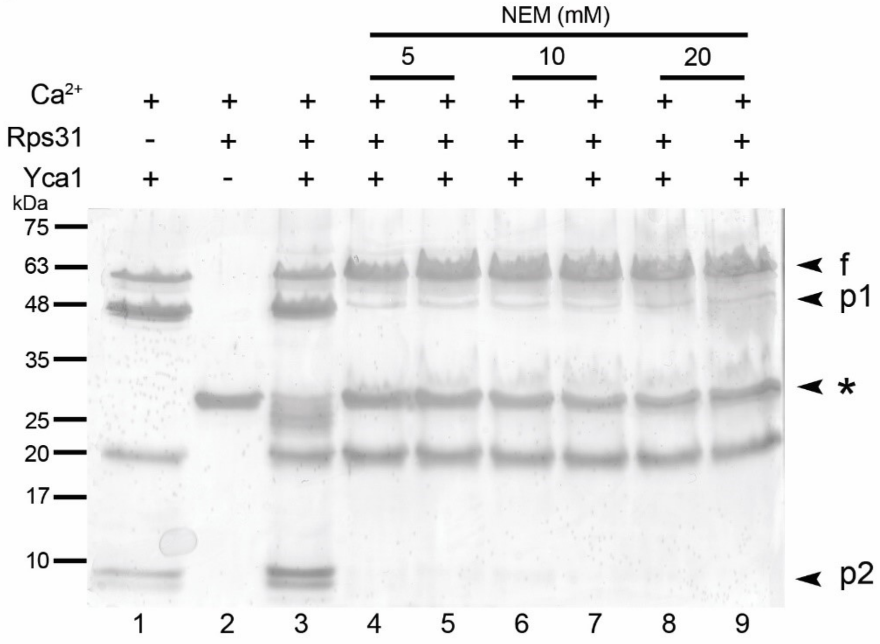

b

NEM modifcation of catalytic cysteines on Yca1 (Scaffold PTM)

MYPGSGRYTY NNAGGNNGYQ RPMAPPPNQQ YGQQYGQQYE QQYGQQYGQQ NDQQFSQQYA
PPPGPPPMAY NRPVYPPPQF QQEQAKAQLS NGYNNPNVNA SNMYGPPQNM SLPPPQTQTI
QGTDQPYQYS QCTGRRKALI IGINYIGSKN QLRGCINDAH NIFNFLTNGY GYSSDDIVIL
TDDQNDLVRV PTRANMIRAM QWLVKDAQPN DSLFLHYSGH GGQTEDLDGD EEDGMDDVIY
PVDFETQGPI IDDEMHDIMV KPLQQGVRLT ALFDSCHSGT VLDLPYTYST KGIIKEPNIW
KDVGQDGLQA AISYATGNRA ALIGSLGSIF KTVKGGMGNN VDRERVRQIK FSAADVVMLS
GSKDNQTSAD AVEDGQNTGA MSHAFIKVMT LQPQQSYLSL LQNMRKELAG KYSQKPQLSS
SHPIDVNLQF IM

c

Site	*Peptide Sequence*	*Modification*	*Localization*	*Ascore*
C176	GcINDAHNIFNFLTNGYGYSSDDIVILTDDQNDLVR	C2 Nethylmaleimide	100%	1000.00
C297	LTALFDScHSGTVLDLPYTYSTK	C8 Nethylmaleimide	100%	1000.00

Supplementary Table S2

Primer ID	Sequence
Yca1-K158A-F	ACTGGGCGTAGAGCGGCTTTGATTATC
Yca1-K170A-F	TACATAGGTTCAGCAAATCAACTGCGT
Yca1-K352A-F	GGTTCTATATTCGCGACCGTTAAGGGA
Yca1-K355A-F	TTCAAGACCGTTGCGGGAGGTATGGGC
Yca1-K384A-F	ATGTTATCAGGTTCGGCGGATAATCAAACTTCT
Yca1-S346A-F	GCTGCTTTGATTGGTGCTTTAGGTTCTATA
Yca1-RFP-SalI-F	TTT*GTCGAC*ATGAAGATGAGCCTCGAAG
RFP-XbaI-R	GCGC*TCTAGA*TTAGGCGCCGGTGG
XhoI-ADH1-F	TTT*CTCGAG*AAGGTGAGACGCGCATAACCG
SalI-ADH1-R	AAA*GTCGAC*GTGATATGAGATAGTTGATTG
SalI-Rsp5-F	TT*GTCGAC*ATGCCTTCATCCATATCCGTC
SmaI-Rsp5-R	TT*CCCGGG*TTCTTGACCAAACCCTATGGT
SmaI-RFP-F	TT*CCCGGG*GCCTCCTCCGAGGACGTC
